# A Nasal Complication of Nasopharyngeal Swab for Reverse Transcription Polymerase Chain Reaction (RT-PCR) Detection of SARS-CoV-2 mRNA

**DOI:** 10.7759/cureus.16183

**Published:** 2021-07-05

**Authors:** Mario Rigante, Pasqualina M Picciotti, Claudio Parrilla

**Affiliations:** 1 Department of Otolaryngology - Head and Neck Surgery, Fondazione Policlinico Universitario A. Gemelli Istituto di Ricovero e Cura a Carattere Scientifico (IRCCS) Università Cattolica del Sacro Cuore, Rome, ITA

**Keywords:** sars-cov-2 mrna, nasopharyngeal swab, covid-19, nasal cavities, complication

## Abstract

Nasopharyngeal (NP) and oropharyngeal (OP) specimens in the detection of the SARS-Cov-2 RNA are considered to have the highest diagnostic sensitivity and they have been recommended by the World Health Organization as the most reliable test. However, collecting NP specimens require specialized operators and adequate technique.

We describe an intranasal breaking of the nasopharyngeal swab for anatomical reasons needing a surgical removing. We conclude that a safely procedure needs possibly a check for septal deviations or other causes of nasal obstruction.

## Introduction

Nasopharyngeal (NP) and oropharyngeal (OP) specimens in the detection of the SARS-Cov-2 RNA are considered to have the highest diagnostic sensitivity and they have been recommended by the World Health Organization as the most reliable test. However, collecting NP specimens requires specialized operators and adequate technique.

Wang et al. reported that OP swabs were used much more frequently than NP ones in China, however, the SARS-CoV-2 RNA was detected only in 32% of OP swabs, which was significantly lower than that of NP (63%) [1]. A COVID-19 investigation team in the US comparing NP and OP specimens found similar results and the US Centers for Disease Control and Prevention (CDC) recommends collecting the upper respiratory NP swab [2].

Different papers have been recently published in order to define the better way to perform a proper nasal and oropharyngeal swab procedure. A correct technique is essential in the screening of COVID-19 infection [3-5].

## Case presentation

With the approval of the ethics committee of the Fondazione Policlinico Universitario A. Gemelli IRCCS Università Cattolica del Sacro Cuore, we describe a case of intranasal breaking of the nasopharyngeal swab for anatomical reasons needing a surgical removing.

**Figure 1:**
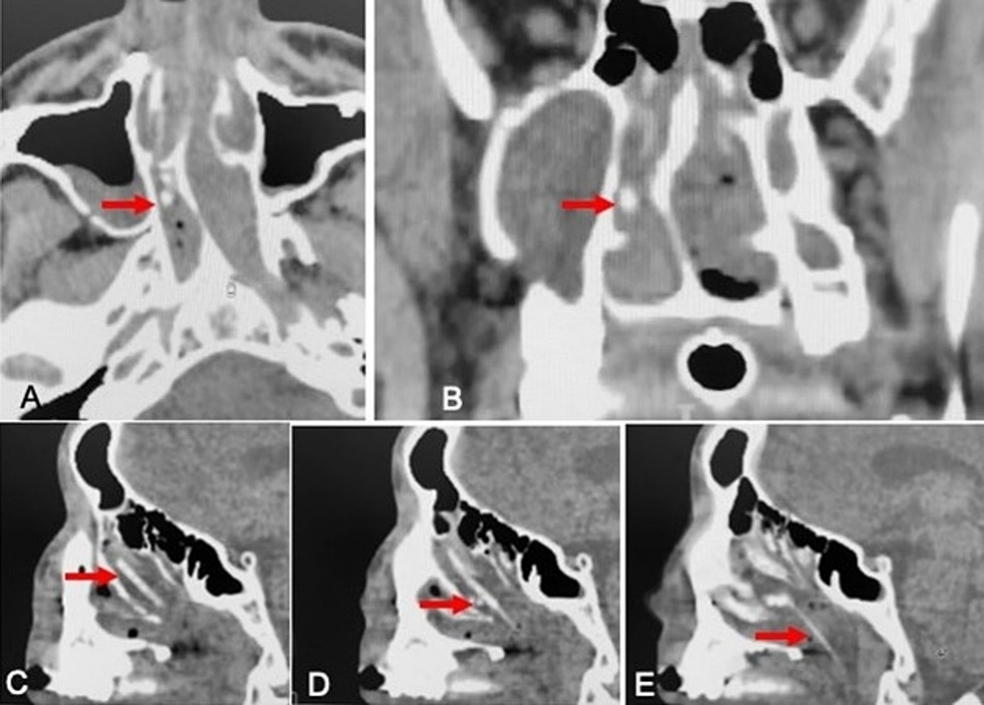
CT scan of the nose CT scan showing the swab (arrow) behind the septal spur in axial and coronal view (A,B) and the entire swab in the sagittal projection (C,D,E) reaching the nasopharynx and after rupture dislocated in the upper part of the nose and also migrated below the middle turbinate.

**Figure 2:**
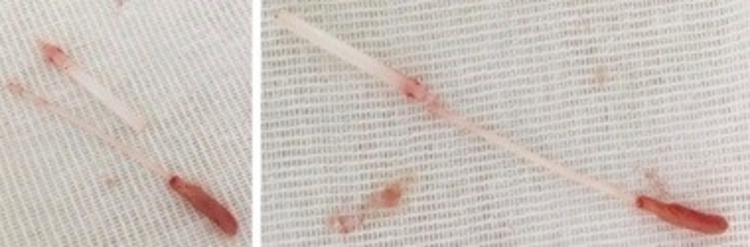
Nasal swab The extracted swab in two parts after performance of septal spur removal.

We hereby report the case of a 32-year-old male patient admitted to the intensive care of trauma center after a motorcycle accident during the pandemic time of COVID-19. OP and NP swabs were performed for SARS-CoV-2 RNA test but during the removal by the healthcare professional, part of the swab broke into the right nasal fossa, with nasal bleeding. The ENT (ear, nose and throat) was referred and the anterior rhinoscopy revealed an important septal deviation into the right with a sub occlusion of the nasal cavity. Therefore, a CT scan was performed for the evaluation of cranial trauma and the swab was located in the upper part of nasal cavity (Figure 1) behind the septal deviation, going down to the nasopharynx. Location was confirmed by nasal endoscopy, but it was not easy to perform because of bleeding and septal deviation that did not allow a complete visualization and extraction of the foreign body. For this we performed a mini-septoplasty, and removal of septal spur was followed by removal of the two swab in two fragment (Figure 2).

## Discussion

The described case confirms that NP swab must be carefully performed as described by other authors for detection of SARS-CoV-2 mRNA avoiding false negative findings [3, 5].

Recent papers by Piras et al. [5] described that nasopharyngeal swabbing could be considered as a trivial procedure, and an inappropriate nasopharyngeal sampling, performed by untrained operators, can cause false negative findings and, it can expose healthcare workers and patients to risks of contagion. Di Maio et al. [3] and Petruzzi et al. [4] also described how to correctly collect sample by nasopharyngeal (NP) swab in order to avoid false negative.

Another subject related to NP swab is possible complications of the procedure. Nasal traumatism related to NP swab can be due to an unfavorable anatomy as we showed. An accurate anterior rhinoscopy is useful to evaluate the opening of the nasal fossa and to guide the operator in the correct execution of the swab. Nasal obstruction can be due to common conditions (septal deviations and/or turbinate hypertrophy) previously evaluated by a physician familiar with the nasal anatomy.

Recently, iatrogenic cerebrospinal fluid fistulas have also been described after nasal swab for COVID‐19 [6-8]. The authors hypothesized that improper technique where the swab is not entered parallel to the palate due to incorrect head tilt increases the risk of complications. As a possible mechanism in fistulas and in our case the swab was not inserted horizontally but in upwards direction. Moreover, it is necessary to be very careful in cases of maxillofacial trauma due to the possible bone modifications that can predispose to important complications.

In our personal experience, we also can argue that septal deviation caused the swab rupture during the removal from the nose, and finally another possible problem is related to the position: our patient was lying on the bed and not sitting.

A final consideration is about the bilaterally swab sampling. There is a significant heterogeneity in guidance for nasopharyngeal swab performance unilaterally or bilaterally. Recent guidelines of the CDC for collecting nasal specimen (Updates on February 2021) [9] described the position head (back 70 degrees), the performance modality until resistance is encountered or the distance is equivalent to that from the ear to the nostril of the patient. These indications also claimed that specimens can be collected from both sides but it is not necessary if a deviated septum or blockage creates difficulty in obtaining the specimen from one nostril. In this condition, the same swab should be used to obtain the specimen from the other nostril.

Our experience confirms this indication: in presence of an apparent septal deviation and or a difficulty introducing the swab, it can be performed only in the more patent nasal fossa, to avoid nasal traumatism and potential swab rupture.

## Conclusions

A safely procedure needs possibly a check for septal deviations, other causes of nasal obstruction or nasal traumatism. A correct procedure should be performed, and when the operator finds obstacle in the insertion of swab in one nostril it should be carried out carefully in the most patent nasal fossa, without forcing or pointing the swab upwards both incoming and outgoing.
